# HIV-1 *Trans* Infection of CD4^+^ T Cells by Professional Antigen Presenting Cells

**DOI:** 10.1155/2013/164203

**Published:** 2013-05-07

**Authors:** Charles R. Rinaldo

**Affiliations:** Department of Infectious Diseases and Microbiology, University of Pittsburgh Graduate School of Public Health, Pittsburgh, PA 15261, USA

## Abstract

Since the 1990s we have known of the fascinating ability of a complex set of professional antigen presenting cells (APCs; dendritic cells, monocytes/macrophages, and B lymphocytes) to mediate HIV-1 *trans* infection of CD4^+^ T cells. This results in a burst of virus replication in the T cells that is much greater than that resulting from direct, *cis* infection of either APC or T cells, or *trans* infection between T cells. Such APC-to-T cell *trans* infection first involves a complex set of virus subtype, attachment, entry, and replication patterns that have many similarities among APC, as well as distinct differences related to virus receptors, intracellular trafficking, and productive and nonproductive replication pathways. The end result is that HIV-1 can sequester within the APC for several days and be transmitted via membrane extensions intracellularly and extracellularly to T cells across the virologic synapse. Virus replication requires activated T cells that can develop concurrently with the events of virus transmission. Further research is essential to fill the many gaps in our understanding of these *trans* infection processes and their role in natural HIV-1 infection.

## 1. Introduction

The uptake of human immunodeficiency virus 1 (HIV-1) by professional antigen presenting cells (APCs) and subsequent transfer of virus to CD4^+^ T cells can result in explosive levels of virus replication in the T cells. This could be a major pathogenic process in HIV-1 infection and development of acquired immunodeficiency syndrome (AIDS). This process of *trans* (Latin; to the other side) infection of virus going across from the APC to the T cell is in contrast to direct, *cis* (Latin; on this side) infection of T cells by HIV-1. Note that this is a narrow definition of *trans* infection, as direct, cell-to-cell spread of virus is a classic phenomenon in viral infections [1], including HIV-1 [2, 3]. In fact, T cell-to-T cell infection involves many factors that are part of APC-to-T cell *trans* infection, including expression of CD4 on T cells and formation of a virologic synapse [4, 5] and evasion of neutralizing antibody [6] and the viral inhibitory effects of antiretroviral treatment (ART) [7, 8]. The use of *trans* infection in this review, however, refers to the *in vitro* model where infection of APC with low levels of HIV-1 leads to replication of virus in T cells, that is, orders of magnitude more than *cis* infection of either APC or T cells, and T cell-to-T cell or APC-to-APC *trans* infections. 

The outcome of the APC-to-T cell *trans* infection process has been considered to be central to sexual transmission of HIV-1 at mucosal (anal and vaginal) and epidermal (foreskin) sites [9–11]. A further, potentially critical feature is its role in progression of HIV-1 infection. In either case, the initial phase of the HIV-1 *trans* infection process involves unique, *cis* interactions and replication cycles of virus in the major types of professional APC, that is, subsets of dendritic cells (DC), monocytes/macrophages, and B lymphocytes. Recent reviews have focused on DC-T cell [12] and macrophage-T cell [13] *trans* infections. This review will focus on the role of each type of APC in HIV-1 *trans* infection, how these infected cells transfer virus to the CD4^+^ T cell, and the outcome of this “kiss of death.” Note that this review does not cover information on *trans* infection of simian immunodeficiency virus (SIV) in nonhuman primates. The reader is referred to recent reviews on SIV infection for this information [14, 15].

## 2. Langerhans Cells (LC), Dermal DC (dDC), and Interdigitating DC (idDC)

### 2.1. *Cis* Infection: Introduction

**Figure 1:**
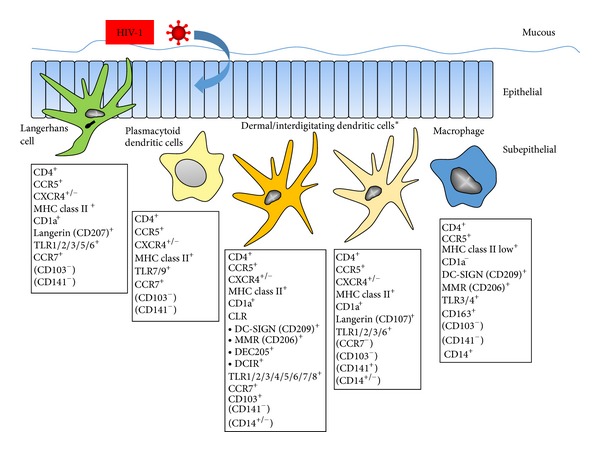
*Major types of human, mucosal, professional APC that serve as targets for primary HIV-1 infection in the mucosa*. Expression of the CXCR4 coreceptor can occur after DC activation *in vivo* and *in vitro* culture. *Note that dDC and idDC are undergoing continuous revision (hence the markers in parentheses) and could represent a continuum of a single type of DC regulated by local environmental influences. CD103 = integrin alpha 3; CD141 = blood dendritic cell antigen 3; CD163 = scavenger receptor.

LC serve among a family of cellular sentries detecting microorganisms that enter the epidermis, and most pertinent to HIV-1, the vagina, ectocervix, and male foreskin [16]. They recognize pathogens through C-type lectin receptor (CLR), Toll-like receptor (TLR), and other pattern recognition receptors [17]. LC express langerin (CD207), a CLR that binds microorganisms for endocytosis, and have Birbeck granules that are involved in endosomal recycling. LC are negative for the DC-specific intercellular adhesion molecule-3-grabbing nonintegrin (DC-SIGN; CD209) CLR. LC are therefore primarily distinguished from dDC and idDC in that the latter are langerin negative and express DC-SIGN. Note that in this review, idDC refers to the conglomeration of tissue and interstitial myeloid DC, which are understudied in HIV-1 infection. These distinguishing properties are important in how DC subsets can interact with HIV-1 and result in *cis* and *trans* infections.  Figure 1 presents basic phenotypic characteristics of LC, dDC, and idDC that could be involved in HIV-1 *cis* and *trans* infection during mucosal transmission. The complexity of human DC is becoming more evident, as detailed in a recent, comprehensive study comparing blood and skin DC for a plethora of markers [18].

### 2.2. *Cis* Infection: Early Studies

The first indication that DC were involved in HIV-1 infection was a report in 1984 that patients with AIDS had a significant reduction in the number of LC in the epidermis [19]. This was followed by a series of histopathological studies that either supported [20] or failed to support [21–23] that LC were infected with HIV-1 *in vivo.* There was also some *in vitro* evidence of HIV-1 infection of LC [24]. These early, contradictory findings were related to limitations in the clinical and laboratory study designs and available technologies to detect HIV-1 infected cells *in vivo*. 

By 1996, a comprehensive investigation conducted by Compton et al. [25] was able to quantitate epidermal LC densities in serial biopsies from two burn patients over 11 years after they were infected with HIV-1 via contaminated blood transfusions. Immunostaining for intracellular p24 viral capsid antigen showed that HIV-1-infected LC constituted an extraordinary one-third to one-half of the epidermal LC over many years of asymptomatic infection. Interestingly, the number of epidermal LC in the HIV-1 infected persons was comparable to uninfected persons and occasionally increased. Other studies detecting viral antigen or HIV-1 RNA by polymerase chain reaction (PCR) supported that LC are infected *in vivo* with HIV-1 [26–28]. Epidermal LC isolated from AIDS patients have a range of 107 to 3,645 HIV-1 DNA copies per 10^5^ LC, comparable to that found in CD4^+^ T cells [29]. However, on a direct cellular basis, the relative frequency of idDC harboring HIV-1 DNA from spleens of AIDS patients ranges from 1/720 to 1/18,000, which is 10–100 times less than CD4^+^ T cells (range: 1/17 to 1/190) [30]. Nevertheless, it is clear that a significant number of LC and idDC are targeted by HIV-1 *in vivo* during untreated HIV-1 infection. 

Similar to the improved *in vivo* evidence, data from upgraded *in vitro* models reinforced that LC can be *cis* infected with HIV-1. LC purified from migrating cells of human epidermis explants express CD4, the primary receptor for HIV-1 [31], and coreceptor CCR5 [32, 33], which is required for infection by R5 tropic strains of HIV-1 [34]. Consequently, cultured LC can be productively infected with R5 HIV-1 [35].

### 2.3. *Cis* Infection: HIV-1 Receptors

**Figure 2:**
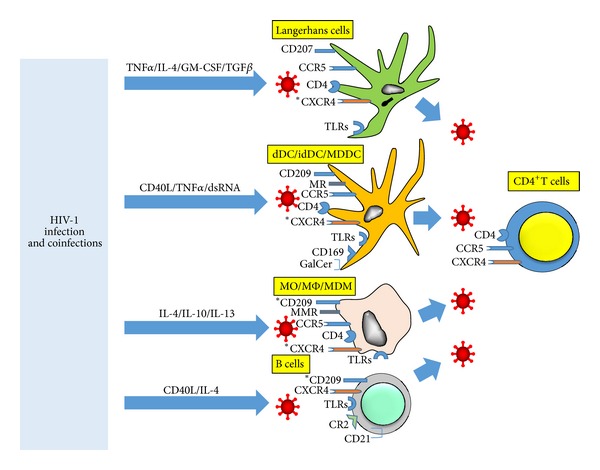
Factors that enhance *cis* infection of APC also enhance *trans* infection. Inflammatory cytokines produced in response to HIV-1 infection and co-infections enhance various stages of HIV-1 infection of APC, including maturation of DC and activation of B cells. This in turn is essential for transfer of HIV-1 to CD4^+^ T cells and conferring them with the ability to produce prodigious amounts of HIV-1. *CXCR4 and CD209 expressed on activated APC.

Expression of the primary CD4 receptor and chemokine coreceptors for HIV-1 in dDC and idDC differs as to their mucosal, dermal, and tissue sites, cytokine/chemokine milieu, and study design (e.g., *in situ, ex vivo,* and *in vitro*) [36] (Figure 2). Thus, early studies failed to detect expression of CD4 on freshly isolated dDC [37], while others showed intense CD4 expression on tonsil idDC subsets [38]. Indeed, the distinction between dDC and idDC is further blurred in that various subsets of dDC appear to migrate to and populate draining lymph nodes [39]. Evidently CD4 is expressed on various if not all myeloid DC populations *in vivo*, although there is little understanding of its function besides serving as the primary receptor for HIV-1 [40]. 

The expression of CD4 and CCR5, as well as CLR, on LC in endocervical mucosa and foreskin is purported to be a basis for the preferential sexual transmission of R5 HIV-1 [41–44]. The dDC and idDC also express these HIV-1 receptors, although there are little phenotypic data on these human APC. Steady state LC do not constitutively express the other major HIV-1 coreceptor, CXCR4, *in vivo*, but do so after *in vitro* culture [33] (Figure 2). Coreceptor expression is upregulated by cytokines IL-4 and TGF-*β* [45] and GM-CSF [46]. In addition, LC derived by treatment of CD34^+^ neonatal cord blood stem cells with cytokines (GM-CSF, IL-4, tumor necrosis factor *α* (TNF-*α*), and TGF-*β*) express CD4 and both CCR5 and CXCR4 and can be productively infected with R5 and X4 tropic HIV-1 [45, 47]. HIV-1 replication can be further enhanced by treatment of such cultured LC with recombinant CD40L [48]. These studies support that activated LC, and likely dDC and idDC, can occur *in vivo* through interactions with activated CD4^+^ T cells expressing cytokines and CD40L and are thereby rendered permissive for both R5 and X4 HIV-1 infection [48].

Additional evidence supporting the conventional pathway for *cis* infection of LC by either R5 or X4 strains is that this can be blocked by pretreatment of the cells with natural and synthetic ligands for CCR5 (RANTES; regulated on activation, normally T cell-expressed and secreted) or CXCR4 (SDF-1; stromal cell-derived factor 1) [49, 50], as well as blockers of gp41 fusion [51]. Moreover, LC from persons who are heterozygous for the 32 base pair deletion mutation in CCR5 ORF (delta 32/wt) are less susceptible to HIV-1 infection than LC from CCR5 ORF wt/wt individuals [35]. 

Activation of LC due to the local cytokine milieu could explain how both CCR5 and CXCR4 are expressed on cervical DC [52]. Furthermore, these cytokines can be induced in LC in response to sexually transmitted infections. Yeast (*Candida albicans*) and bacterial (Gram negative and positive organisms and their products) infections act as agonists for TLR2/4/5 in vaginal and skin explants and also induce TNF*α* production [53, 54]. This enhances susceptibility of LC to HIV-1 *cis* infection. TLR triggering leads to lower levels of the HIV-1 restriction factor, apolipoprotein B mRNA-editing, enzyme-catalytic, polypeptide-like 3G (APOBEC3G), in LC. Interestingly, this phenomenon is not demonstrable in monocyte-derived DC (MDDC). 

Herpes simplex virus type 2 (HSV-2), a common sexually transmitted pathogen, enhances both HIV-1 *cis* infection of LC and *trans* infection of T cells by LC [55]. This could be related to the protective effect of langerin against HIV-1 *cis* infection of LC [56], in that HSV-2 competes with HIV-1 for attachment to langerin and also induces a decrease in langerin expression. Interestingly, this effect does not require full cycle replication of HSV-2 in the LC, as it can be mediated by UV-inactivated virus and the TLR3 ligand, poly I:C (synthetic double stranded RNA). 

Notably, these studies used different bodily sources and laboratory methods to isolate and develop LC *in vitro* which likely result in variable properties including state of activation. Early studies indicated that *cis* infection of APC can vary as to the source of HIV-1 and *in vitro* conditions [57], which is still being reestablished in the recent literature [58]. Thus, for example, LC captured from epidermal sheets of suction blisters have different phenotypic and functional properties after *in vitro* culture [59]. Such epidermal-derived LC can be infected with either R5 or X4 strains of HIV-1 and transmit these viruses to T cells within tonsillar tissue explants [60]. Intracellular CXCR4 present in LC [33] is expressed externally through an activation mechanism during *in vitro* processing. This could explain how CXCR4 is expressed on freshly isolated epidermal LC and that these cells can be infected with X4 HIV-1 [46]. Likewise, activated LC derived from CD34^+^ stem cells can efficiently *trans* infect T cells with X4 HIV-1 [61]. This also suggests that lack of CCR5 expression by LC in some studies [62] could be due to vagaries of the *in vitro* model. 

Infection of LC and dDC with HIV-1 has undergone revisionist changes in recent years as different DC subsets have been recognized. Using the human skin explant model, Kawamura and colleagues [63] demonstrated that 2 distinct DC subsets emigrate upon culture, that is, CD1a^+^langerin^+^DC-SIGN^−^LC and CD1a^+^ langerin^−^DC-SIGN^−^dDC, as well as CD1a^−^langerin^−^DC-SIGN^+^ dermal macrophages (Figure 1). CD1a is a transmembrane glycoprotein used to classify phenotypes of tissue DC. It is similar in structure to MHC class I and can heterodimerize with *β*-2 microglobulin and present lipids to T cells. Only LC could be productively infected with HIV-1 R5. Differences in HIV-1 infection of LC subsets could be related to their levels of activation. Indeed, under steady-state conditions, RNA transcriptome profiles of LC are indicative of less inflammatory, migratory, and T cell stimulatory activity, as well as greater expression of cell adhesion and retention molecules, than dDC [64]. More complexity in DC subsets and HIV-1 infection is evident in that dDC include a CD14^+^ subpopulation [65] that appears permissive for productive HIV-1 infection [66].

Recent studies provide further evidence that the susceptibility of LC to HIV-1 *cis* infection and productive replication is dependent on their state of maturation [11, 56]. Immature LC bind and take in HIV-1 via langerin. However, they do not support productive HIV-1 infection, as virus taken up by langerin is internalized into Birbeck granules and destroyed. This protective effect can be overcome by adding larger amounts of HIV-1 to the LC cultures, whereby immature LC become productively infected [67]. Such a natural barrier to HIV-1 infection could be highly significant in preventing HIV-1 transmission. This dichotomy in *cis* infection of LC with HIV-1 based on langerin and the extracellular concentration of infectious HIV-1 has been challenged by evidence that HIV-1 efficiently infects LC *in vitro* [68]. Moreover, it is not clear how infection of LC with low levels of HIV-1 can result in *trans* infection of T cells if virus is destroyed within the LC. One explanation of this paradox is that there is an altered, lower expression of langerin due to activation of LC [61]. This allows virus to enter the LC without being destroyed and traffic to CD1a-containing, multivesicular bodies, where clusters of virions are transmitted to T cells. Once infected by HIV-1, immature LC also become more mature and consequently are more competent in activating CD4^+^ T cells [69]. This effect on LC maturation could thereby enhance *trans* infection.

Besides the X4/R5 paradigm of infection patterns of HIV-1, enhanced susceptibility of vaginal LC to infection with the subtype E clade of HIV-1 has been proposed as the basis for a predominance of heterosexual transmission in Thailand [70]. However, this result was not reproducible [71].

HIV-1 *cis* infection of LC as well as idDC can be blocked by anti-HIV-1 neutralizing and nonneutralizing antibodies. This involves the Fc *γ* receptor for IgG and IgA, implicating this process in protection of mucosa from HIV-1 infection [68]. 

### 2.4. *Trans* Infection

Early evidence from cocultures of epidermal sheets with T cell lines first suggested that LC can *trans* infect T cells with HIV-1 [72]. There were concurrent findings that blood-derived DC could *trans* infect T cells with HIV-1 [73], which are detailed later. This principle regarding LC was confirmed and extended by Pope et al. [74] using an *in vitro* skin explant model to show presence of stable, nonproliferating conjugates of DC (presumably including LC) and memory CD4^+^ T cells. Addition of HIV-1 to these mixed DC-T cell cultures resulted in production of virus by 4 days. It was subsequently formally demonstrated by Ayehunie et al. [75], using the basic DC-T cell coculture model still employed today, that HIV-1-infected LC (and also blood purified DC) transmit virus to uninfected CD4^+^ T cells. Current principles of DC-T cell *trans* infection established by this early, pioneering study were (a) cocultivation of LC and DC isolated as monocyte-negative, low density cells, with allogeneic CD4^+^ T cells results in massive virus production; (b) *trans* infection requires a short, 30 min cell-cell contact; (c) proteolytic striping of cell surface-bound virus from the LC or DC does not decrease the efficiency of virus transmission, suggesting that internalized virus rather than plasma membrane-bound virus is required for *trans* infection; (d) effective virus transfer by LC or DC requires prior activation of T cells; (e) LC or DC mediate *trans* infection of T cells at least 100-fold more efficiently than T cell-to-T cell *trans* infection. The authors concluded that LC and DC could play a key role in the dissemination of virus to T cells they encounter in skin or lymphoid tissue, a hypothesis that is still prevalent almost 20 years later. 

We now know that LC can mediate *trans* infection of CD4^+^ T cells with both R5 and X4 strains of HIV-1 [49]. This occurs in LC-T cell clusters, primarily involving proliferating, CD4^+^ memory T cells [51]. Using a foreskin explant model, it was shown that HIV-1 initially forms virologic synapses between keratinocytes and LC [76]. Virus is soon found within migrating LC in the inner foreskin, with presence of LC-T cell conjugates where there was evidence of T cell *trans* infection. 

With the establishment of the LC-T cell *trans* infection model, it became clear that various aspects of *cis* infection of LC described earlier significantly affect their capacity to *trans* infect T cells. Thus, it was first noted that treatment of LC with anti-CD4 receptor Ab, coreceptor ligands, and antiviral agents had no effect on *trans* infection of T cells [47]. This suggested a second pathway to the CD4/CCR5 receptor, productive infection, where LC capture HIV-1 by other receptors and transmit the virus to T cells. However, later evidence supported that entry via CD4/CCR5 receptors on LC leads to *trans* infection of CD4^+^ T cells [35]. This is supported by the blocking of HIV-1 *trans* infection from LC by inhibitors of R5 coreceptor binding and gp41 fusion [77]. Hence, there could be at least 2 pathways by which HIV-1 *trans* infects T cells via LC, as is detailed later.

Finally, intriguing data from a hallmark study by Hladik et al. [78] using a vaginal mucosal explant system supported a different pathway model for HIV-1 *trans* infection by LC. They showed that HIV-1 R5 infection was first established independently in both intraepithelial CD4^+^ T and LC through a CCR5 binding pathway. HIV-1 fused with and entered T cells, resulting in productive *cis* infection, whereas uptake by LC involved endocytosis and nonproductive sequestration of virus particles for up to 3 days. Furthermore, virus particles were observed in membrane synapses between the LC and T cells that emigrated from the epithelium. This study therefore provided rare, *in situ* evidence supporting the virus synapse between DC and T cells as the principal site of HIV-1 *trans* infection. 

## 3. Blood Myeloid DC

### 3.1. *Cis* and *Trans* Infection *In Vivo*

A key to our understanding of the role of myeloid DC in both *cis* and *trans* infection with HIV-1 R5 and X4 is direct examination of HIV-1 interactions with primary DC and T cells in blood and tissues of HIV-1 infected persons. As with LC, it is difficult but possible to delineate HIV-1 infection in primary DC examined *ex vivo*. Thus, a portion of myeloid DC in blood of untreated HIV-1 infected persons is positive for proviral HIV-1 DNA, but at much lower levels than CD4^+^ T cells [79]. Once on long term, virus suppressive ART, proviral DNA, and viral particles are not detectable in myeloid DC or other types of circulating DC [80]. It is much more difficult, however, to directly prove *trans* infection of endogenous virus from such blood or tissue DC *in vivo*. The usual approach therefore has been to examine blood myeloid DC *in vitro* for their capacity to support *cis* and *trans* infection with HIV-1. 

The first study to definitively demonstrate HIV-1 *trans* infection *in vitro* was over 20 years ago using blood myeloid DC [73]. DC were purified by density gradient centrifugation from peripheral blood mononuclear cells (PBMCs) that were depleted of T cells, B cells, monocytes, and natural killer (NK) cells. These immature DC did not support *cis* infection with HIV-1 X4 virus but resulted in explosive virus replication when mixed with polyclonally activated, autologous CD4^+^ T cells. Replication of virus was noted within T cells clustered around the DC. Coincident with this breakthrough discovery was a controversy as to whether such blood DC could actually support productive HIV-1 *cis* infection. Results from several research groups showed high levels of HIV-1 *cis* replication in cultures of purified blood DC [81–84]. However, using similar blood-derived, myeloid DC models, others found that DC did not support HIV-1 *cis* infection yet could result in high levels of *trans* infection of CD4^+^ T cells [85]. This could relate to the amount of input virus, as productive *cis* infection was noted in later studies using purified blood CD11c^+^ myeloid DC and high titers of input R5 or X4 HIV-1 [86]. The integrin CD11c is commonly used to distinguish phenotypes of tissue DC and is involved in their cell-cell adhesion. Importantly, viral replication occurs in a small percentage of circulating CD1c^+^ (BDCA-1^+^) myeloid DC that express CD4 and low levels of CCR5 [87]. These virus-infected DC can *trans* infect CD4^+^ T cells. Thus, these data using a “natural” form of myeloid DC derived directly from blood suggest their potential for supporting both HIV-1 *cis* and *trans* infection *in vivo*. 

Many of the characteristics of *trans* infection in these early DC studies still pertain to our current understanding. Thus, *trans* infection could (1) be blocked by antibodies to CD4 and T cell accessory molecule CD80 [85], (2) be augmented by activated CD4^+^ T cells expressing CD40L [85], proinflammatory cytokines such as IL-2 [88] and nominal antigens [89, 90], and (3) result in extensive, apoptotic death of the T cells within the DC-T cell clusters [89, 91]. However, *cis* infection of DC was becoming more complex. Although it appeared to require CD4 [90] and R5 or X4 coreceptors [92], as for HIV-1 infection of LC, there was accumulating evidence that HIV-1 infected the DC by more than one pathway. Electron and light microscopy studies suggested that HIV-1 could enter DC by several routes—fusion with the plasma membrane, endocytosis in coated pits, and phagocytosis [93, 94]. 

Digging deeper into the pathways of DC infection with HIV-1, Cameron et al. [95] showed that after *in vitro* exposure to HIV-1, viral DNA was more frequent in purified, blood CD11c^+^ myeloid DC from HIV-1 negative donors than monocytes or resting CD4^+^ T cells. Infection was more efficient with R5 than X4 strains of HIV-1. Importantly, infection of CD11c^+^ DC-T cell cocultures with R5 virus resulted in productive, *trans* infection of CD4^+^ T cells, whereas *cis* infection of these DC with HIV-1 was inefficient. 

In 2000, Geijtenbeek et al. [96] reported that myeloid DC expressed the CLR DC-SIGN, which served as a receptor on MDDC for HIV-1 *trans* infection. Clearly the key to delineating the importance of DC-SIGN expression in both HIV-1 *cis* and *trans* infection is whether these *in vitro* findings using MDDC correspond to *in vivo* activity. Initial evidence showed that blood DC express very little DC-SIGN and that DC-SIGN is expressed on a greater proportion of dermal DC and idDC. Further proof is that DC-SIGN-expressing mononuclear cells are found in Peyer's patches of gut mucosa [97, 98]. Indeed, 1%–5% of these cells in the rectal mucosa from non-HIV-1 infected adults are DC-SIGN^+^ compared to only 0.01% of blood mononuclear cells [99]. Gurney et al. [100] established that although gut DC express CD4, CD11c and HLA DR, they do not express CCR5 or CXCR4. Interestingly, DC-SIGN is expressed on CD14^+^ cells of the same mucosal region. This was an early indication that steady state, tissue macrophages express DC-SIGN, in contrast to blood monocytes and monocyte-derived macrophages (see later). 

Most important is that R5 HIV-1 preferentially binds to the HLA DR^+^ DC-SIGN^+^ mononuclear cells isolated from the gut mucosa and is efficiently transferred to and replicates in CD4^+^ T cells [100]. This *trans* infection can be blocked by mannan and anti-DC-SIGN mAb. Interestingly, gut mucosa mononuclear cells in HIV-1 infected subjects express more DC-SIGN than HIV-1 uninfected persons. 

#### 3.1.1. Role of Regulatory T Cells (Treg) in *Trans* Infection

An early suggestion that Treg could be involved in the expression of DC-SIGN was the strong, positive correlation of the IL-10/IL-12 ratios (mRNA) and DC-SIGN cell numbers in the gut tissues. Notably, Treg are an amalgamation of several varieties of naturally resident and pathogen-induced CD4^+^ T cells [101]. These regulatory cells can inhibit DC and T cell activation and thereby decrease DC-mediated HIV-1 *trans* infection [102, 103]. 

A major Treg pathway involves generation of adenosine from extracellular adenosine triphosphate (ATP) and adenosine diphosphate that is mediated by ectoenzymes CD39 and CD73 on Treg membranes [104]. Adenosine binds to the A2A receptor leading to increased cyclic adenosine monophosphate that inhibits T cell differentiation and proliferation. This provides the negative feedback necessary to prevent tissue damage that may result from a persistent inflammatory state. However, it has been noted that extracellular ATP also dampens DC-T cell *trans* infection [105]. This ATP effect was linked to lower numbers of *trans*-infected T cells with both X4 and R5 HIV-1 and had no effect on *cis* infection of the T cells. Evidence suggested that extracellular ATP led to increased lysosomal degradation of internalized virus in the DC. Clearly we need more information on the potential role of Treg and adenosine in HIV-1 *trans* infection.

#### 3.1.2. Effect of HIV-1 Infection on Capacity of DC to Mediate HIV-1 *Trans* Infection

A critical point not usually addressed in *trans* infection studies is whether *in vivo* infection with HIV-1 alters the capacity of DC to mediate *trans* infection. Most of this evidence is suggestive and indirect. Nevertheless, various aspects of the number and functional capacity of blood and tissue myeloid DC could bear on their ability to mediate *trans* infection. Primary, blood myeloid DC from persons with untreated, progressive HIV-1 infection exhibit low T cell stimulation and cytokine/chemokine production [106]. In fact, circulating myeloid DC levels decline very early in blood during acute HIV-1 infection [107]. Pertinent to *trans* infection is that myeloid DC directly circulating in blood of untreated HIV-1 infected persons are partially activated [108], possibly due to direct stimulation by HIV-1, but still undergo maturation after stimulation *in vitro *by TLR ligation. Interestingly, these partially activated DC accumulate in lymph nodes of the infected persons, supporting their potential to mediate *trans* infection. Such blood myeloid DC also exhibit abnormally low production of the inflammatory cytokines IL-6, TNF-alpha, and IL-12 *in vitro* [109]. However, this depends on the type of stimulation, as DC from HIV-1 infected subjects produce high levels of cytokines and chemokines in response to stimulation with TLR7/8 agonists [107]. They also retain their ability to stimulate allogeneic T cell responses and upregulate DC maturation markers. Contrasting studies show a defect in circulating myeloid DC early in HIV-1 infection, including poor antigen presentation and production of cytokines [110]. Finally, the very low expression of DC-SIGN on blood myeloid DC does not differ in relation to progression of HIV-1 infection [111]. 

Of special interest is that HIV-1 derived, uridine-rich ssRNA serves as a ligand for TLR7/8 [112], through which it upregulates programmed cell death 1 ligand 1 (PD-L1; CD274) expression on myeloid DC. PD-L1 in turn is an inhibitory signal on DC which triggers PD-1 on CD8^+^ T cells, thereby impeding their reactivity [113]. This pathway could impact on HIV-1 *trans* infection and requires further study.

#### 3.1.3. HIV-2 *Trans* Infection

HIV-2 infection is much less pathogenic than HIV-1 infection [114]. Interestingly, mature MDDC cannot *trans* infect autologous CD4^+^ T cells with either R5 or X4 tropic HIV-2 [115]. This block could be due to poor *cis* infection of DC with HIV-2. It points to the possibility that a lower capacity to mediate *trans* infection could be involved in slower progression of HIV-1 infection.

## 4. MDDC and CD34^+^ Cell-Derived DC

### 4.1. *Cis* and *Trans* Infection

The major breakthroughs in our understanding of HIV-1 *cis* and *trans* infection stem from discovery of simple methods for deriving large numbers of myeloid DC from CD34^+^ bone marrow stem cells using various growth factors [116] and more importantly from blood monocytes using IL-4 and GM-CSF [117]. These *in vitro* derived DC are presumed to mirror differentiation of myeloid DC *in vivo* and have many of the properties of blood and tissue dDC and idDC. Indeed, the bulk of our knowledge of HIV-1 *trans* infection is based on MDDC derived from peripheral blood monocytes by *in vitro* culture with IL-4 and GM-CSF. This is because of (1) the ease of accessibility and processing, and relative low cost, of deriving large numbers of MDDC from CD14^+^ blood monocytes, as well as (2) many phenotypic and functional traits of MDDC that are common to natural myeloid DC circulating in blood. Supporting evidence that HIV-1 *cis* and *trans* infection mediated by MDDC is a significant gauge of HIV-1 pathogenesis is that differentiation of monocytes into CD1a^+^ DC *in vitro* correlates with low CD4^+^ T cell counts and high viral loads in HIV-1 infected adults [118]. Nevertheless, similar to studies using LC, dDC, and idDC, there are significant differences among techniques used in studies of *trans* infection in the MDDC-T cell model that could result in discordant results. These include different strains of HIV-1, purification and maturation techniques for MDDC, and types of CD4^+^ T cell targets (e.g., autologous and allogeneic; continuous cell lines). In particular, several differences in the pathways of HIV-1 *cis* infection in MDDC are critical in defining the down-stream events of *trans* infection. 

Basic principles of both *cis* and *trans* HIV-1 infection of these DC were soon demonstrated and are pertinent today. First, despite early conflicting studies [119, 120], it became apparent that both MDDC and CD34^+^ precursor cell-derived DC are poorly permissive for productive HIV-1 *cis* infection [121–123]. The *cis* infection process requires CD4, which is more efficient via cell associated virus [124] and can be enhanced by virus opsonized with antibody and complement [125, 126], while blocked by IgG alone via binding to FC*γ*R [127]. However, the fate of IgG-opsonized virus depends on several factors including time of exposure to the MDDC and the opsonization pathway used to enter and traffic within the cells [128]. Complement, that is, C5a, can also be triggered in HIV-1 infected T cells, drawing in immature MDDC and resulting in enhanced *trans* infection [129].

#### 4.1.1. DC Maturity Significantly Affects HIV-1 *Trans* Infection

The dogma that has emerged over the years is that immature DC in submucosal tissues (see earlier) capture HIV-1 and taxi it to local draining lymphoid tissues, where they undergo maturation that enhances their antigen presenting properties [130]. These mature DC also are most efficient at *trans* infection of T cells. Indeed, early studies supporting efficient HIV-1 *trans* infection by immature DC [131, 132] were not confirmed, as mature DC appear more effective at *trans* infection of CD4^+^ T cells [133, 134]. This could, however, be dependent on the form of DC maturation. MDDC matured with CD40L, TNF*α*, and bacterial lipopolysaccharide (LPS) have a respective, decreasing propensity to support HIV-1 *cis* infection, while all 3 types of mature MDDC promote efficient *trans* infection [134]. Moreover, mature MDDC and purified blood myeloid DC display increased uptake of HIV-1 and survival of the engulfed virus [133]. Thus, MDDC that are treated with CD40L, or different combinations of LPS, IFN-*γ* and double stranded synthetic RNA (poly I:C), produce large amounts of IL-12 (termed (DC-1) that polarize CD4^+^ T cells to differentiate into type 1 helper (Th1) cells [135]. In contrast, MDDC treated with prostaglandin E2 (termed DC-2) promote the induction of type 2 helper (Th2) cells [136]. The DC-1 mediate more efficient *trans* infection than DC-2 [137].

Early studies indicated that immature DC support greater *cis* infection by HIV-1 than mature MDDC [131, 138, 139]. However, this was challenged by further work showing that *cis* infection was dependent on R5 and X4 tropism and the type of DC maturation agent used. Thus, both immature and mature MDDC show poor fusion and *cis* infection by X4 virus [140]. This is not due to differential expression of coreceptors, as immature MDDC express similar amounts of CXCR4 and CCR5 [141]. Indeed, immature MDDC can utilize both tyrosine kinase (Src and SyK) [142] and the protein kinase A and C [143] pathways in supporting HIV-1 *cis* infection. Nevertheless, immature MDDC are less susceptible to *cis* infection by X4 compared to R5 HIV-1, which is related to less efficient fusion by X4 virus to immature MDDC. Furthermore, *trans* infection mediated by mature DC with R5 but not X4 virus is enhanced by CXCL12, the chemokine ligand for CXCR4. This could effectively select for R5 virus infection that predominates in the earlier phase of natural HIV-1 infection [144].

A key issue is that, similar to HIV-1 infection of LC, HIV-1 infection of MDDC can inhibit the maturation process [103, 145]. This includes lack of stimulation of allogeneic T cell responses and expression of cell surface maturation molecules such as MHC class II and CCR7.

Of significance is that *trans* infection from mature DC to T cells is dependent on levels of CD4, as blocking CD4 on immature MDDC enhances their *trans* infection with X4 virus [146]. This could be due to redirecting of CD4 biasing virus entry into DC through DC-SIGN and subsequent efficient *trans* infection of T cells. Mature MDDC also have enhanced HIV-1 endocytosis and are highly effective at concentrating HIV-1 in virologic synapses [147]. 

#### 4.1.2. Cholesterol and Tetraspanins Play a Key Role in HIV-1 *Trans* Infection

Cholesterol-enriched surface domains of DC are central to their capacity to mediate *trans* infection [133]. These are concentrated in lipid rafts, which are cholesterol-sphingolipid floating microdomains involved in cholesterol transport, endocytosis, and signal transduction [148]. The rafts contain HIV-1 receptors including DC-SIGN [149, 150]. Hence, it is not surprising that cholesterol and lipid rafts are involved in HIV-1 binding, entry and subsequent intracellular trafficking in DC [151, 152] which is required for HIV-1 *trans* infection. Moreover, lipid rafts are linked to the actin cytoskeleton and microtubules, whose integrity is also essential for HIV-1 *trans* infection [153]. Evidence for a role of cholesterol in HIV-1 *trans* infection is that enhancing cholesterol efflux in DC by triggering of regulators of cholesterol metabolism, that is, peroxisome proliferator-activated receptor gamma and liver X receptor, decreases HIV-1 *trans* infection [154]. Furthermore, increasing the level of cellular cholesterol by inhibiting the cholesterol transport protein ATP-binding cassette A1 (ABCA1) in these DC restores their ability to mediate HIV-1 *trans* infection [154]. 

Interestingly, host-derived lipids embedded within HIV-1 virions are involved in capture of the virus by DC and subsequent *trans* infection of T cells. In fact, alpha 2-3 ganglioside GM3 can mediate Env-independent binding to DC through its sialic acid residue [155]. This effect is accentuated in mature DC through sialyllactose in the ganglioside [156]. This process of virus capture by mature DC involves binding of sialic acid-binding Ig-like lectin 1 (Siglec-1, CD169) on the surface of DC to sialyllactose-containing gangliosides on the viral membrane [157]. This provides HIV-1 *trans* infection pathway mediated by mature DC that is independent of CLR.

Intriguing recent reports indicate that IFN-*α* acts to inhibit viral infection by reducing sterol metabolic activity [158]. This involves the oxysterol cholesterol-25-hydroxylase, which catalyzes the oxidation of cholesterol [159], thereby altering the cell membrane and inhibiting virus fusion, including HIV-1 [160]. It would be of interest to examine a possible role of this innate immune pathway in inhibiting HIV-1 *trans* infection.

Mature DC concentrate captured HIV-1 in late endosomal vesicles containing CD63 and CD81 tetraspanins, which are not found in immature DC. In fact, it is apparent that HIV-1 particles colocalize within DC in tetraspanin-rich compartments that include CD9, CD63, CD81, and CD82 [161, 162]. Some of these tetraspanins, particularly CD63, are carried in the viral membrane upon budding from the DC. These data point to the well-documented role of tetraspanins in *cis* infection of T cells [163, 164] and support a role for tetraspanin-rich microdomains in HIV-1 *trans* infection via egress of virus from DC to T cells. 

### 4.2. DC-SIGN Receptor

MDDC express the CLR DC-SIGN, which is a major receptor for HIV-1 *cis* and *trans* infection [96]. Thus, HIV-1 *trans* infection by MDDC can be blocked by antibodies to DC-SIGN [96], mannose-binding lectin [165], and interfering RNA specific for DC-SIGN [166, 167]. Virus is being transmitted across a virologic synapse between the DC and T cell after cell-to-cell contact [168]. This unique discovery opened a new, non-CD4/coreceptor dependent pathway for HIV-1 *cis* and *trans* infection of T cells by passaging through DC. DC-SIGN-expressing DC retain the ability to *trans* infect T cells for several days and traffic towards M-tropic (R5) but not T-tropic (X4) HIV-1 [169]. 

The end result of these early studies was still the finding of poor *cis* replication of either R5 or X4 HIV-1 in DC. Instead of the conventional fusion and entry by CD4/coreceptors in T cells, carbohydrate regions of gp120 bind to DC-SIGN [170], which is in a tetrameric form [171]. A combination of N-linked glycosylation sites on gp120 are involved in HIV-1 binding to DC-SIGN [172, 173]. Notably, DC-SIGN also functions as a pattern recognition receptor for nonself glycans [174]. Virus entry into DC via DC-SIGN or other CLR does not lead to processing of HIV-1 antigen for MHC class I or II presentation [175]. Internalization motifs on the cytoplasmic tail of DC-SIGN trigger endocytosis and uptake of virus, which ends up in low pH nonlysosomal compartments. Leukocyte-specific protein 1 binds to the cytoplasmic region of DC-SIGN and directs internalized virus to the proteasome [176]. Importantly, downregulation of DC-SIGN by microRNA-155 indicates that this CLR is under tight host control that could affect binding of gp120 and uptake of virus [177]. 

Glycosylation sites on gp120 are key to binding to DC-SIGN, and thus viruses differing in the V1V2 and V3 variable loops of gp120 are differentially captured by DC-SIGN expressing cells [178]. In fact, R5 virus from patients with end-stage AIDS that does not undergo the switch to X4 virus has reduced ability to bind DC-SIGN [179]. This is due to a lack of potential N-linked glycosylation sites in the gp120 in these late-stage R5 variants. Following entry, HIV-1 sequesters in DC and remains available in an infectious form for *trans* infection [180, 181]. In fact, MDDC can support infectious R5 virus [182] but not X4 virus [183] for several weeks in culture.

The efficiency of *cis* infection of DC and subsequent *trans* infection also involves TLR8 signaling stimulated by HIV-1 encoded ssRNA and DC-SIGN signaling stimulated by gp120 [184]. This initiates transcription of integrated proviral DNA by RNA polymerase II and generation of full length viral transcripts.

R5 viruses are preferentially transmitted to T cells by DC [185], which is not blocked by anti-HIV-1 neutralizing antibodies [186]. Likewise, antibody neutralized, dual-tropic HIV-1 is captured by DC-SIGN and Fc receptors on immature MDDC and can *trans* infect T cells [187]. Of significance is that *trans* infection can occur in both activated and resting T cells [188] but requires activated T cells for extensive virus replication. Indeed, *trans* infection is enhanced during antigen-specific activation of T cells by DC, leading to selective destruction of anti-HIV-1 T cells [189]. Further evidence of the importance of DC-SIGN to *trans* infection is the enhanced binding to DC-SIGN by complement-opsonized virus, leading to its uptake and *trans* infection of T cells by immature MDDC [190]. Of note is that uptake of complement-bound virus also involves several integrins [191].

### 4.3. Dual Pathways of HIV-1 *Cis* Infection of DC Affect *Trans* Infection

Data from the earliest investigations of DC-SIGN-expressing MDDC began to clarify our understanding that there are both nonproductive and productive pathways of HIV-1 *cis* infection of DC that determine the efficiency of *trans* infection [192]. The bulk of the evidence supports that there are 2 parallel, distinct pathways that have different temporal phases within the DC. The first 24-hour phase of HIV-1 infection is a nonproductive pathway following binding to DC-SIGN and endocytosis. Initiation of this process is enhanced by direct contact of DC with HIV-1 infected cells [124] via HIV-1 Env-mediated virion endocytosis [193], which in this case may not involve DC-SIGN [194]. Endocytosis of cell-associated HIV-1 by DC involves activity of matrix metalloproteinase-9 [195], a collagenase that could be involved with cytoskeletal movements during endocytosis. 

**Figure 3:**
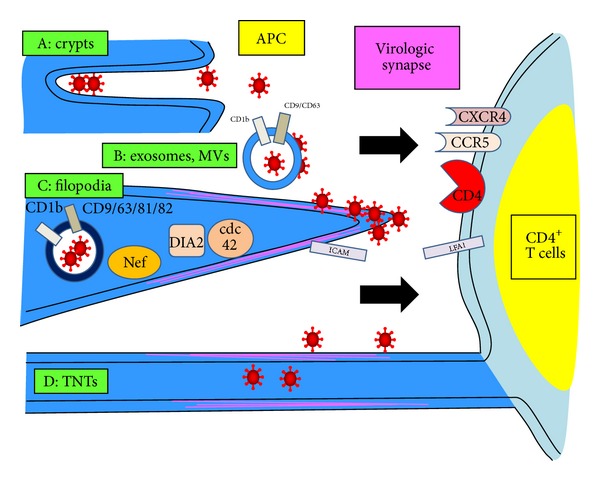
APC crypts, exosomes/microvesicles (MVs), filopodia, and tunneling nanotubes (TNTs) are associated with HIV-1 *trans* infection of T cells. HIV-1 infected APCs enhance *trans* infection of T cells by transferring (a) infectious virus within crypts (extracellular invaginations), (b) release of infected exosomes that express CD1b and tetraspanins, and transporting virus harbored within endosomal MVs expressing CD1b and tetraspanins; (c) viral Nef and cellular dia2 and cdc42 proteins rearrange actin filaments in the infected APC membrane to form filopodia; virus buds from the tips to form the virus synapse with the T cells, which involves ICAM/LFA-1 adhesion molecules and CD4/CCR5 (CXCR4) viral receptors. (d) Virus is transported through within TNT conduits and on the outside (“surfing”) of TNTs directly into T cells.

HIV-1 infection of DC by the endocytic process is resistant to proteolysis by trypsin [196]. Both viral endocytosis and subsequent *trans* infection by the DC can be decreased by HIV-1 protease inhibitors [197]. Virus accumulates in nonlysosomal, mildly acidic, endocytic compartments that are rich in DC-SIGN and conducive to maintaining infectivity [198, 199]. Virus is transferred to a virologic synapse formed between the DC and the CD4^+^ T cell (Figure 3). 

The second phase of infection is where a smaller fraction of HIV-1 enters DC by CD4 receptor/coreceptor-mediated membrane fusion [200]. DC-SIGN could also play a role in this process by interacting with gp120 and thereby increasing the Env binding site for CD4 [201]. By 2 days, this nonendosomal virus undergoes productive, complete cycle replication that is also transmitted via a virologic synapse to T cells. 

HIV-1 is therefore transmitted from DC to T cells in two time-dependent phases. This explains how a higher expression DC-SIGN on immature MDDC resulted in non-productive, *cis* infection [202], yet these MDDC could transfer virus efficiently to T cells. HIV-1 infected, mature MDDC also activate CD4^+^ T cells, facilitating the process of *trans* infection [69]. Further distinguishing the two phases of HIV-1 infection in DC is that they differ in mRNA expression, particularly in decreased cathepsin activity during the replicative virus pathway [203]. This could enhance HIV-1 replication by avoiding lysosomal enzymatic degradation.

The endocytosis pathway for *trans* infection from DC to T cells has been challenged [204]. Thus, the Raji B cell line engineered to express endocytosis-defective DC-SIGN is as effective at mediating *trans* infection as cells expressing wild-type DC-SIGN [205]. Moreover, immature MDDC required virus fusion and production of infectious virions to *trans* infect T cells. Others have shown that mature DC concentrate virus particles in nonendosomal, intracellular compartments [206] or on the surface of DC [207].

An intriguing offshoot of the HIV-1-DC endocytic *trans* infection pathway is that infectious virus particles that are taken into endosomes can be exocytosed and productively infect T cells as an alternate *trans* pathway [208]. Similar results for efficient *trans* infection not requiring intact DC have been reported using fractionated membranes or microsomes from HIV-1-infected, dead DC and other cell types [209]. The exosomes express HLA-DR1, MHC-like transmembrane glycoprotein CD1b, and transpanins CD9 and CD63, which are incorporated upon release of virus from the endosomes. Moreover, uptake of such exosomes is enhanced in mature DC, which can then *trans* infect T cells in a unique manner independent of HIV-1 envelope glycoprotein [210]. 

The vagaries of the *in vitro* DC-T cell model used to assess HIV-1 *trans* infection are evident when comparing these latter results of efficient infection via cell-associated and exosome-associated virus to whole, apoptotic T cells. Thus, feeding of apoptotic, uninfected, activated CD4^+^ T cells to DC can inhibit both HIV-1 *cis* and *trans* infection [211]. While these apoptotic cells actually mature the DC, they also induce virus inhibitory APOBEC3G.

### 4.4. Non-DC-SIGN Receptors

Antibodies to DC-SIGN and DC-SIGN ligands do not always block *cis* infection of DC [202], nor do they always block *trans* infection [212]. Thus, HIV-1 *trans* infection is not consistently linked to DC-SIGN expression [213–216]. Indeed, circulating blood myeloid DC express little DC-SIGN yet can *trans* infect T cells [99]. This suggests that HIV-1 *trans* infection of CD4^+^ T cells can occur via DC-SIGN-independent mechanisms. Most of these molecules, however, work similar to DC-SIGN by binding carbohydrate residues on gp120, concentrating virus on the cell surface and enhancing virus uptake by DC. Thus, other CLRs can be involved in DC-mediated *trans* infections. These include DC immunoreceptor (DCIR) [217], which is expressed on all DC subsets [218]. However, the role of DCIR in *trans* infection is complicated by findings that DCIR expression is induced in CD4^+^ T cells by HIV-1 infection and that this is also evident as a paracrine-like effect in uninfected CD4^+^ T cells [219]. Moreover, these DCIR-expressing T cells exhibit enhanced replication of HIV-1. The virus acts on tyrosine and threonine residues of the DCIR immunoreceptor tyrosine-based inhibitory motif, associated with activation of various kinase pathways [220].

Syndecan-3, a DC-specific heparan sulfate proteoglycan, captures HIV-1 through binding of gp120 and thereby enhances both *cis* and *trans* infection by MDDC [221]. Surfactant protein A, a collectin family molecule involved in pathogen recognition in the lungs, amniotic fluid, and vaginal tract, enhances HIV-1 *trans* infection at low, acidic pH similar to the vaginal tract [222]. Finally, the glycosphingolipid galactosyl ceramide (GalCer) is a mucosal epithelial receptor for HIV-1 that binds gp41 and is also expressed on immature MDDC and primary myeloid from blood and mucosal tissue [223]. Blocking with anti-GalCer mAb inhibits HIV-1 *trans* infection. 

## 5. Role of CD4^+^ T Cells in DC-Mediated HIV-1 *Trans* Infection

### 5.1. CD4^+^ T Cell Subsets

The efficiency of the APC-to-T cell *trans* infection pathway is dependent on the type and metabolic state of the CD4^+^ T cell. There is a complexity in how CD4^+^ T cells relate to *trans* infection based on their tissue location and activated, inflammatory state, which has received little attention. CD4^+^ T helper (T_H_) cells come in many varieties ranging from proinflammatory (T_H_1) to anti-inflammatory (T_H_2) and beyond [224]. Similar to T cell plasticity involved in anticancer immunity [225], the subset and functional capacity of T_H_ cells in virus infection can vary as to the organ tissue and pathogen encountered. Recently it was reported that follicular memory CD4^+^ T cells defined as CXCR5^+^PD-1^+^Bcl-6^+^ are a major compartment for HIV-1 infection in viremic hosts [226]. The intimate interaction in lymph nodes of APC with follicular CD4^+^ T cells [227] points to these being major targets for *trans* infection. These tissue T cells secrete IL-21 that regulates T cell, B cell, and NK cell differentiation and function [228].

Eradication of HIV-1 infected T cells could require recognition and lysis by cytotoxic T lymphocytes (CTLs) [229]. Here we enter the complex world of HIV-1 *cis* infection of professional APC that is required for induction and maintenance of anti-HIV-1 CTL, as well as *trans* infection of CD4^+^ T cells. Indeed, a new, potential aspect of *trans* infection of CD4^+^ T cells that should be explored involves its role in the quest for a true or functional cure of HIV-1 infection [230, 231]. ART and immunotherapies that inhibit HIV-1 *cis* infection may not inhibit HIV-1 *trans* infection. Different CD4^+^ T cell subsets, in particular the resting central and transitional memory subsets known to harbor the latent HIV-1 reservoir during ART [232], could be involved in HIV-1 *trans* infection. Stealth, low level passage of virus between APC and these T cells in lymph nodes and other tissues could be a source of virus persistence in the face of ART.

### 5.2. Membrane-to-Membrane Contact Is Essential for *Trans* Infection

Direct contact of infected DC with CD4^+^ T cells forms a virologic synapse that is central to *trans* infection [233]. Several elegant microscopy studies have shown that infection of DC by HIV-1 induces dramatic changes in the actin cytoskeleton that enhance DC-T cell interaction [234]. Thus, HIV-1 infection of immature DC activates the membrane cell division control protein 42 homolog which is a regulator of actin polymerization, leading to filopod extensions of the DC cytoplasmic membrane [235]. These filopodia are also dependent on Nef and the formin protein diaphanous 2 that induce polymerization of actin [236]. The filopodia bear a large amount of budding HIV-1 particles on the tips of their long extensions [236]. Filopodia also protrude from CD4^+^ T cells and express CD4 that is involved in transfer of HIV-1 virions from DC [237].

### 5.3. T Cell Activation Is Essential for *Trans* Infection

Highly productive HIV-1 *trans* infection requires activated CD4^+^ T cells. Indeed, many studies have used polyclonally activated, allogeneic T cells and continuous T cells lines which presumably undergo further DC-mediated allostimulatory activation. Under conditions of natural infection within the host, HIV-1 effectively takes advantage of the immunologic synapse, that is, MHC-antigen-T cell receptor complex, to form a virologic synapse. This is supported by mathematical models that recapitulate the dynamics of HIV-1 infection, where simultaneous priming and infection of T cells by DC is predicted to drive early HIV-1 infection [238]. Alternatively, the virologic synapse could form by interactions among roaming T cells and tissue-resident or blood DC, without an antecedent, antigen recognition, DC-T cell interaction. Simple contact of uninfected, immature DC can augment HIV-1 replication in infected, resting CD4^+^ T cells [239]. This occurs in the absence of nominal antigen presentation. However, the basis of this phenomenon is unclear. For example, DC without nominal antigen do not alter HIV-1 proviral DNA in resting CD4^+^ T cells [240]. Moreover, many of the T cells within the DC-T cell clusters die by apoptosis [241].

DC infected with HIV-1 can express nominal viral antigens that prime T cells *in vitro*. However, this immunologic process activates the P38MAPK/STAT3 pathway resulting in upregulation of T cell inhibitory molecules in the DC including cytotoxic T-lymphocyte antigen (CTLA-4), TNF-related apoptosis-inducing ligand (TRAIL), lymphocyte-activation gene-3 (LAG3), and T-cell Ig mucin-3 (TIM-3) [242]. Thus, it is possible though not yet proven that during T cell priming by infected DC, HIV-1 *trans* infection could be diminished. 

### 5.4. Central Role of the Virologic Synapse in *Trans* Infection

The virologic synapse is where critical virus transmission events occur between the DC and T cell involving conventional HIV-1 CD4 and chemokine receptors on the T cells. Thus, *trans* infection is accentuated by interaction of various cell adhesion molecules such as LFA-1/ICAM-1 [243] which are upregulated on activated mature DC and are involved in the DC-T cell virologic synapse [137] (Figure 3). HIV-1 *trans* infection is impaired in MDDC from patients with leukocyte adhesion deficiency type 1 whose T cells lack expression of LFA-1 [244]. There are contradictory data, however, that not all LFA-1 ligands play this role, as ICAM-1 [243] and ICAM-3 [245] have not been confirmed to be involved in HIV-1 *trans* infection. However, additional evidence that such binding is important in *trans* infection is that it can be prevented by inhibitors that block the virus-cell fusion process, for example, CD4 attachment blockers (mAb), fusion blockers [T20], and chemokine receptor blockers (RANTES) [246, 247].

Various intracellular processes are operative in forming the DC-T cell virologic synapse that is required for efficient *trans* infection. Activation of Rho guanine nucleotide-exchange factor LARG in DC after HIV-1 infection is one such factor involved in formation of virus-T cell synapses [248]. During the DC-T cell adhesion process, HIV-1 translocates across the synapse to form virus particles in intracellular compartments in endocytic vesicles of the T cells [2]. Although there is enhanced replication of R5 virus in this microenvironment [185, 249], both R5 and X4 viruses are transmissible to the T cells. 

Importantly, the CD4^+^ effector-memory T cell (Tem) subset is most susceptible to *trans* infection by R5 HIV-1, which is related to expression of the CCR5 coreceptor [250]. In contrast, CD4^+^ naïve T cells (Tn) are more susceptible to *trans* infection with X4 virus in relation to expression of CXCR4.

The efficiency of *trans* infection is further dependent on expression of HIV-1 *nef* [233, 251]. Nef enhances this process by promoting HIV-1 *cis* infection of immature DC and recruitment of T cells to DC [252]. Nef heightens HIV-1 *trans* infection by upregulating DC-SIGN expression on DC and promoting DC-T cell clustering [146], while enhancing activation and proliferation of CD4^+^ T cells [253]. However, recent work has found that Nef induces tetherin, a type 2 integral membrane protein that can inhibit budding of virus in HIV-1-infected immature DC [254]. Thus, there appear to be competing inhibitory and enhancing effects of Nef on HIV-1 *cis* infection of DC that need to be clarified.

### 5.5. Neutralizing Antibodies Can Enhance *Trans* Infection

In contrast to early studies suggesting that *trans* infection could be blocked by neutralizing antibodies [255], later investigations showed that HIV-1 *trans* infection was protected from such effects [187]. In fact, neutralizing antibodies enhance the ability of both immature and mature MDDC to* trans* infect T cells with X4 virus [256], suggesting that this process could be involved in the switch from R5 to X4 that occurs during natural, *in vivo* HIV-1 infection. These seemingly conflicting results could be related to the viral target of the antibody. Thus, neutralizing antibody directed against gp41 is much more effective at blocking *trans* infection than that directed to gp120 [257]. This is postulated to be due to binding of gp41-specific antibody to the DC plasma membrane prior to the formation of a virologic synapse, positioning them to inhibit virus transfer. 

## 6. NK Cells and DC-Mediated HIV-1 *Trans* Infection

NK cells have bidirectional interactions with DC where they promote maturation of DC and production of cytokines that activate T cells [258, 259]. This process could therefore bear on HIV-1 *trans* infection. Indeed, NK cells drive DC maturation [260]. As discussed earlier, mature DC mediate enhanced HIV-1 *trans* infection of T cells. Some NK cells express CD4 and are susceptible to HIV-1 *cis* infection. Infection of NK cells by HIV-1 has been controversial, but accumulating data support that a CD4-expressing NK cell subset is permissive for HIV-1 replication [261]. Moreover, HIV-1 infected DC are resistant to NK cell-mediated killing or “editing” due to enhancement of antiapoptotic factors in the DC [262]. This could thereby indirectly augment HIV-1 *trans* infection mediated by DC. Additional studies are needed to better define NK cell interactions with DC and T cells in relation to HIV-1 *trans* infection.

## 7. Coinfections and DC-Mediated HIV-1 *Trans* Infection

### 7.1. Bacteria

Given the importance of DC maturation and T cell activation in both *cis* and *trans* infection, it is logical that coinfections could affect these processes. This has been studied in the context of an enhancing role of genital coinfections on HIV-1 *trans* infection in sexual transmission. Bacterial vaginosis is a mildly symptomatic condition, for example, discharge, odor, and pain, occurring when the predominant commensal lactobacilli in the vaginal tract are largely replaced by Gram negative or positive bacteria [263]. It is associated with increased risk for HIV-1 infection [264]. Mucosal fluids from women with bacterial vaginosis activate and mature MDDC [265], although a more recent study has failed to show that this leads to enhanced HIV-1 *trans* infection [266]. Nevertheless, maturation of genital tract DC could be due to the effects of Gram negative bacterial LPS [135], which also enhances their ability to mediate HIV-1 *trans* infection [131, 267]. This likely occurs at least in part through binding of LPS to TLR4 [268]. TLR2 triggering of DC, which includes bacterial peptidoglycan and lipoproteins ligands, also enhances HIV-1 *trans* infection [269]. Finally, some combinations of oral Gram negative bacteria activate the HIV-1 promoter and viral replication in immature DC as well as macrophages [270]. This supports a potential role for polybacteria activating HIV-1 infection in the oral cavity.

A second context of bacterial coinfection enhancing HIV-1 *trans* infection is through translocation of bacterial products across the gastrointestinal (GI) tract to the blood, which leads to systemic immune activation. Indeed, there is extensive evidence for early, enhanced HIV-1 replication in resident CD4^+^ T cells of the GI tract, with lysis of the infected cells and associated disruption of the epithelial-blood barrier [271]. Lamina propria CD4^+^ T cells are under a steady state of activation and express the primary CD4 receptor and CCR5 and *α*4*β*7 coreceptors, making them fertile ground for either *cis* or *trans* infection. Moreover, recent studies have revealed that there is selective destruction of the Th17 cell subset of CD4^+^ T cells in the GI tract [272]. These cells produce IL-17 as well as IL-22 and IL-26 and are important in antimicrobial defense. Lamina propria mononuclear cells including CD1c^+^ DC are also constitutively activated in relation to chronic TLR signaling by microbial products [273]. These DC enhance productive infection of Th17 cells after activation by *E. coli* [274]. This is related to MHC class II-restricted Ag presentation to the T cells by the DC. 

Tuberculosis is the leading cause of death in HIV-1 infected persons [275]. Although macrophages are the APC primarily targeted by *Mycobacterium tuberculosis* (MTB), infection of DC is also notable [276]. MTB binds to DC-SIGN by mannose-capped lipoarabinomannan in its cell wall [277]. Soluble factors produced by MTB-infected macrophages lead to partial maturation of DC, with further production of immune mediators by the DC [278]. This macrophage-DC inflammatory milieu is associated with increased HIV-1 *trans* infection by the DC. Microscopy studies indicate that MTB-HIV-1 coinfection of DC decreases degradation of the virus, resulting in greater sequestration of HIV-1 in the intracellular *trans* infection pathway [279]. 

### 7.2. Fungi

Several diverse types of fungi induce maturation of DC and coincidental enhancement of HIV-1 *trans* infection. Thus, infection of MDDC with* Candida albicans* augments HIV *trans* infection of T cells in relation to increases in expression of CD80 and CD86 on the DC [280]. *Cryptococcus neoformans* and *Penicillium marneffei*, both of which can be lethal to HIV-1 infected persons, enhance DC-mediated HIV-1 *trans *infection in association with increases in DC-T cell conjugates and CD4^+^ T cell activation [281, 282]. 

### 7.3. Parasites

Coinfection of malarial parasites with HIV-1 is common in coendemic areas [283]. Infection with* Plasmodium falciparum* increases HIV-1 viral load and enhances progression to AIDS [284]. The enhancing effect of malaria on DC-mediated *trans* infection could be due to hemozoin, a pigment that is released in excess after destruction of the infected erythrocytes. The hemozoin-loaded DC are partially activated, leading to increased activation and *trans* infection of CD4^+^ T cells [285, 286].

In contrast to enhancement of HIV-1 *trans* infection by malarial parasites, leishmania parasites inhibit *trans* infection by DC [287]. The mechanism of this phenomenon is not clear, although it could be due to competition for binding to DC-SIGN.

## 8. Blocking of HIV-1 DC-Mediated *Trans* Infection by Microbicides and Antiviral Drugs

Although much of this review is premised on the hypothesis that DC serve as the major, initial sites of HIV-1 infection in the mucosa, it needs to be acknowledged that this is a controversial concept. Indeed, there is extensive evidence that CD4^+^ T cells and not DC are the first, primary targets in HIV-1 and SIV sexual transmission models [288]. Other studies, however, support an important role for DC as initial targets in sexual transmission [289] as well as other modes such as mother-to-child transmission [290]. 

HIV-1 captured on spermatozoa via heparan sulfate can efficiently *cis* infect MDDC *in vitro* [291]. Vaginal and ectocervical derived DC also serve as efficient targets for HIV-1 *cis* infection *in vitro* [292]. However, seminal plasma could be a natural barrier to HIV-1 *trans* infection, as it can inhibit this process *in vitro*, possibly by blocking HIV-1 binding to CLR on the DC by high-mannose N-linked carbohydrates [293]. Also, there is increasing evidence of the importance of cross-talk between non-DC mucosal cells in regulating DC mediated *cis* and *trans* infection. Thus, uterine epithelial cells secrete transforming growth factor *β* that decreases DC-SIGN expression on immature DC, consequently inhibiting *trans* infection by the DC [294]. In contrast, genital mucosal epithelial cells also produce thymic stromal lymphopoietin that activates DC [295]. This results in increased HIV-1 *trans* infection of activated CD4^+^ T cells. 

DC infected at these mucosal transmission sites likely spread HIV-1 through *trans* infection of regional CD4^+^ T cells. However, systemic infection with HIV-1 could require trafficking of HIV-1 infected DC to draining lymph nodes. Recent evidence in a humanized mouse model indicates that HIV-1-infected CD4^+^ T cells are very motile, circulating to lymph nodes where they tether to other T cells to form virologic synapses that facilitate cell-to-cell transmission [296]. Similar studies need to be done to assess the role of DC and other professional APC in HIV-1 trafficking and dissemination. 

Based on the premise that DC are early targets during sexual transmission of HIV-1, there has been a good deal of interest in assessing inhibition of HIV-1 *trans* infection by microbicides and antiviral drugs. *Trans* infection by both R5 and X4 viruses can be at least partially blocked by many different nonnucleoside reverse transcriptase inhibitors (NNRTIs) [297]. Others have reported that RTIs such as azidothymidine do not inhibit *trans* infection [196]. Both *cis* infection of DC and DC-mediated *trans* infection of T cells can be inhibited by protease inhibitor saquinavir [298]. Several HIV-1 entry inhibitors targeting Env (BMS-C, T-1249) or CCR5 (CMPD167) have been shown to block *cis* infection of DC [299]. 

Various experimental microbicides act through different inhibitory pathways to decrease HIV-1 *cis* and *trans* infection, some of whose mechanisms of action are still unclear. These include amphibian-derived antimicrobial peptides [300], HIV-1 integrase strand transfer inhibitors [301], Miltefosine, a phospholipid drug that induces type I IFN in DC [302], SAMMA, a polymer derived from mandelic acid [303], HIV-1 fusion inhibitor T-1249 [304], the nematode C-type lectin Mermaid, a structural homologue of DC-SIGN [305], sulfonated polyanion PRO 2000 [306], a soluble CD4-linker-DC-SIGN fusion protein [307], Dendron 12, a multimeric glycomimetic DC-SIGN ligand [308], Inmunoferon, a natural glycoconjugate [309], carbohydrate-binding plant lectins [310, 311], oligomannose glycans [312], trimannosides [313, 314], and advanced glycation end products [315].

## 9. HIV-1 *Trans* Infection Mediated by Plasmacytoid DC (pDC)

In 1999, Siegal et al. [316] defined a population of lymphoid-derived DC in blood, termed pDC, that were highly potent producers of IFN-*α* in response to HIV-1 and other viral infections. The pDC were distinct from myeloid DC in morphology and phenotype, clarifying previous work indicating that a natural IFN-producing cell with a dendritic cell phenotype was the major producer of IFN-*α* [317, 318]. The pDC differ phenotypically from myeloid DC primarily by lack of expression of CD11c and positive expression of the IL-3 receptor, CD123. It is now well established that pDC are central to innate immunity to viral infections through viral DNA and RNA signaling of TLR9 and TLR7, respectively. We also know that pDC have important yet enigmatic roles in both combating and enhancing HIV-1 infection [319]. 

It appears that pDC are less permissive for productive, *cis* infection with HIV-1 than are myeloid DC, even though they both express CD4 and HIV-1 coreceptors [86, 320]. Nevertheless, several studies indicate that pDC derived from blood [95, 321] and thymus [322, 323] can support productive HIV-1 infection with X4 or R5 strains, particularly if the cells are prestimulated with CD40L [324]. Moreover, although pDC do not express DC-SIGN *in vitro* [98], there is evidence of expression of this CLR on pDC subsets *in vivo* [97].

Given these data on expression of HIV-1 receptors and productive *cis* infection of pDC, it is not surprising that pDC can *trans* infect T cells with R5 and X4 HIV-1 [95, 324, 325]. This infection preferentially targets antigen-responding CD4^+^ T cells [325]. Interestingly, productive *cis* infection of the pDC is not required for *trans* infection of T cells with HIV-1 [325].

The ability of pDC to mediate *trans* infection with HIV-1 has been challenged by work showing that, regardless of their state of activation, pDC derived from the same donors as myeloid DC did not *trans* infect their T cells [326]. This is explained at least in part by the antiviral effects of the prodigious amounts of IFN-*α* produced by the pDC. Clearly more information is needed to define whether pDC are able to *trans* infect T cells with HIV-1.

## 10. HIV-1 *Trans* Infection Mediated by Monocytes and Macrophages

Circulating monocytes and their tissue-transformed macrophages are APC that can mediate HIV-1 *trans* infection of CD4^+^ T cells similar to DC [13]. This was first reported in 1990 [327], where blood monocyte-derived macrophages (MDMs) transmitted HIV-1 to peripheral blood lymphocytes, presumably CD4^+^ T cells. Two basic principles of HIV-1 *trans* infection were established with this pioneering study, that is, that antigen-specific activation of the T cells results in greater *trans* infection by the MDM and that anti-CD4 and anti-gp120 antibodies block *trans* infection. It took years for this phenomenon to be extended, as in 1999 Carr and colleagues [328] reported that MDM transmitted HIV-1 to CD4^+^ T cells much more efficiently than *cis* infection of T cells. This process required cell contact, occurred in both allogeneic and autologous systems, and was enhanced by preactivation of the T cells. Overall, *trans* infection mediated by MDM appears as efficient as that mediated by myeloid DC.

### 10.1. *Cis* Infection

#### 10.1.1. HIV-1 R5 and X4 Receptors on Monocytes/Macrophages

As with DC, the efficiency of HIV-1 *trans* infection mediated by monocytes and macrophages is largely dependent on the pathways and proficiency of preceding *cis* infection in these APCs. Given that blood-derived monocytes/macrophages express CD4 and CCR5, but not CXCR4, they are highly susceptible to R5 HIV-1, albeit with much lower production of infectious virus than *cis* infection of CD4^+^ T cells [329]. However, some X4 variants are able to infect monocytes/macrophages. The efficacy of HIV-1 infection largely depends on variations in binding capacity of HIV-1 Env to these coreceptors [330]. It is also apparent that, as with tissue DC and LC, subsets of tissue resident macrophages express both HIV-1 coreceptors *in vivo *[62, 331, 332]. Tissue macrophages, including those within the genital mucosa and foreskin, are consequently potentially susceptible to *cis* infection with either R5 or X4 strains of HIV-1 [17, 41, 44, 292]. HIV-1 infection of monocytes/macrophages is further complicated by evidence that circulating monocytes in HIV-1 infected persons harbor many different phenotypes of HIV-1 [333]. These include HIV-1 variants that use multiple combinations of coreceptors that differ from virus in the corresponding CD4^+^ T cells. 

Interestingly, semen contains enhancers of HIV-1 *cis* replication. For example, prostatic acidic phosphatase (PAP) in seminal fluid forms amyloid fibrils that enhance HIV-1 infection of macrophages [334].

The overall outcome is that *cis* replication efficiency, that is, production of infectious virus, varies greatly among both R5 and X4 HIV-1 strains but is relatively poor in monocytes/macrophages compared to T cells [335]. This is based on the well-documented yet still controversial process of HIV-1 replication in monocytes/macrophages compared to CD4^+^ T cells. In contrast to CD4^+^ T cells, HIV-1 resides and accumulates in specialized compartments within monocytes/macrophages, where new virions form and are eventually released to the extracellular environment [336]. 

#### 10.1.2. TLR Triggering in Monocytes/Macrophages

Monocytes and macrophages express a variety of TLRs depending on their blood and tissue distribution [337]. These can serve as triggers for monocyte/macrophage activation and, hence, affect HIV-1 *cis* infection. Thus, the TLR2 ligand PAM3CSK4, a synthetic triacylated lipopeptide mimic of the acylated amino terminus of bacterial lipopeptide, activates HIV-1 transcription in macrophages [338]. This effect can be counteracted and suppressed by nuclear receptor signaling through, for example, the glucocorticoid receptor. Such nuclear receptor signaling also inhibits the proinflammatory response induced by PAM3CSK4 that activates HIV-1 expression in macrophages. Furthermore, HIV-1 may code for its own activation in macrophages, as suggested by single-stranded RNA of HIV-1 inducing uridine-rich TLR 7/8 ligands that stimulate MyD88-dependent monocyte activation [337].

#### 10.1.3. Regulation of *Cis* Infection in Monocytes/Macrophages

Monocytes are more resistant to productive HIV-1 infection than macrophages, which has been associated with presence of apolipoprotein B mRNA editing enzyme, catalytic polypeptide-like 3A (APOBEC3A) [339]. This cytidine deaminase may inhibit HIV-1 replication in monocytes through an IFN-*α* activated deamination of viral DNA [340]. Also, recent genome-wide scanning has revealed that expression of the gene DYRK1A, encoding a kinase that phosphorylates serine and threonine residues, is associated with efficient *cis* replication of HIV-1 in MDM [341]. 

Macrophages have been divided into M1 and M2 subsets distinguished by their polarized function, with M1 being activated by proinflammatory factors such as IFN-*γ*, TNF-*α*, and LPS, and M2 being activated by anti-inflammatory agents including IL-4, IL-10, and IL-13 [342]. Polarizing cytokines affect HIV-1 replication efficiency in macrophages; IFN-*γ* and TNF-*α* induce activation of the M1 subset of MDM, with downregulation of CD4 and consequent resistance to HIV-1 infection [343]. IFN-*γ*-activated M1 cells can, however, maintain low levels of both R5 and X4 HIV-1 DNA and low virus replication [344]. Regarding M2 macrophages, IL-4-activated M2 cells exhibit resistance to HIV-1 replication at a later, postentry stage. There also are a number of other host proteins and microRNAs known to regulate various stages of HIV-1 *cis* infection of monocyte/macrophages that are beyond the scope of this review [345]. 

An exciting, recently discovered mechanism by which macrophages restrict HIV-1 replication is that they express the dideoxynucleotide hydrolase SamHD1 [346, 347]. The actual mechanism of action is not fully clear but involves inhibition of reverse transcription of HIV-1 DNA. Furthermore, the IFN-inducible protein viperin (virus inhibitory protein, endoplasmic reticulum-associated, interferon-inducible) that was first discovered in macrophages [348] inhibits HIV-1 replication [349]. All of these host factors are likely contributing to the low level replication and persistence of HIV-1 infection, as well as its relative noncytopathic effect, in monocytes/macrophages.

Finally, the phenomenon of autophagy has in recent years been shown to be central to HIV-1 *cis* infection of monocytes/macrophages and therefore likely to affect *trans* infection of T cells. Autophagy is a catabolic process where cellular components are degraded by lysosomal machinery and thereby has a critical role in antigen processing by APC [350]. Although autophagy also functions as an intracellular host defense mechanism against viruses, it can be usurped by HIV-1 infection, particularly in macrophages and CD4^+^ T cells [351]. Whereas autophagy is enhanced in bystander, uninfected CD4^+^ T cells by X4 or R5 Env, leading to apoptosis, uninfected macrophages are resistant to this process [352]. In HIV-1-infected macrophages, the effects of autophagy are more complicated. In fact, early, nondegradative steps in autophagy are required for efficient HIV-1 infection, whereas later, degradative steps are detrimental to HIV-1 replication in macrophages [352, 353].

### 10.2. *Trans* Infection

Monocytes and MDM normally do not express DC-SIGN *in vitro* [96], thus negating this pathway in HIV-1 *cis* and *trans* infection. However, MDM treated *in vitro* with IL-13 [354, 355] or IL-4 [356, 357] express DC-SIGN and, hence, can mediate HIV-1 *trans* infection. M2a MDM derived by IL-4 treatment express DC-SIGN that facilitates *cis* infection with both R5 and X4 HIV-1 and subsequent efficient *trans* infection of T cells [358]. In contrast, M1 MDM derived by TNF-*α* and IFN-*γ* treatment express little DC-SIGN and do not efficiently *trans* infect T cells. 

MDM also express the C-type lectin mannose-binding receptor (macrophage mannose receptor (MMR); CD206) which binds mannosylated carbohydrates on gp120 with high efficiency [359]. Virus binding is consequently inhibited by carbohydrate-binding agents such as mannan, D-mannose, EDTA, and soluble mannose-binding lectin [359], as well as antibody specific for MMR [360]. 

Of particular pertinence to HIV-1 *trans* infection is that certain resident tissue macrophages [97, 361] express DC-SIGN, supporting their potential for mediating HIV-1 *trans* infection. It has been suggested that placental CD163^+^ macrophages (Hofbauer cells) are infected by HIV-1 via their DC-SIGN receptors and that this could represent a pathway for mother-to-child transmission of HIV-1 [362]. However, MMR and not DC-SIGN expression is associated with HIV-1 *trans* infection of T cells by IL-13-treated MDM that express both [354]. Interestingly, IL-13 also inhibits *cis* replication of HIV-1 in MDM [363]. This suggests that *trans* infection mediated by MDM involves other nonreplicative pathways of HIV-1 in these APC. This concept fits with the extensive data that HIV-1 replicates relatively poorly in MDM and tissue resident macrophages [13, 364]. In this regard, the scavenger receptor cysteine-rich protein gp340, which is expressed by macrophages *in vivo* and MDM *in vitro*, is associated with HIV-1 *cis* infection of MDM and their capacity to mediate *trans* infection [365].

Given the ability of MMR to bind HIV-1 with high efficiency, this pathway has been linked to *trans* infection of T cells [359]. Thus, HIV-1 *trans* infection mediated by MDM can be blocked by inhibitors of MMR binding. This process differs, however, from *trans* infection mediated by DC in that virus does not survive beyond 1 day after binding to MDM. Notably, this latter finding contrasts with evidence that HIV-1 survives for weeks within macrophages and can still *trans* infect T cells [366]. Regardless, HIV-1 *trans* infection mediated by monocytes/macrophages is based largely on inefficient *cis* infection of these APC with HIV-1 virions assembling in late endosomes or multivesicular bodies. This involves tetraspanins including CD63 and CD81 that are constitutively expressed in these microvesicles. The virion-containing vesicles are important components in the *trans* infection T cell pathway, where they accumulate at the point of the virologic synapse. Efficient *trans* infection may also involve multiple points of cell contact, termed polysynapses, which are rosette-like structures of an infected macrophage surrounded by many T cells [367]. Finally, expression of the cell adhesion molecule sialoadhesin is upregulated by HIV-1 infection of monocytes, possibly through IFN-*α*, and is related to enhanced *trans* infection [368].

Using the HIV-1-infected MDM model, it is evident that movement of the vesicular body to the virologic synapse is dependent on Gag [369]. HIV-1 forming within these vesicles *trans* infects T cells within 6 hours of cell-to-cell contact [370]. Interestingly, neutralizing antibodies fail to block HIV-1 *trans* infection of T cells mediated by macrophages [371]. Hence, this could function to enhance HIV-1 pathogenesis.

Another intriguing physical pathway for HIV-1 *trans* infection of T cells by macrophages is through filopodial bridges or tunneling nanotubes [372]. Murine leukemia virus can hijack such filopodia that extend long distances between cells [373], suggesting that human retroviruses could use similar conduits. In fact, infection of macrophages with HIV-1 induces the formation of nanotubes that contain have HIV-1 particles [374]. HIV-1 can move rapidly between macrophages via endocytic compartments through these actin-rich, membranous nanotubes [375, 376]. Importantly, nanotube-like formation in HIV-1 infected macrophages is driven by Nef and occurs in both systemic and intestinal lymphoid follicles [377]. These actin-propelled nanotubes serve as conduits for transfer of Nef to B cells, where it inhibits immunoglobulin G2 (IgG2) and IgA class switching. It is not yet known if this is involved in macrophage-to-T cell HIV-1 *trans* infection.

### 10.3. HIV-1 *Trans* Infection in Human Colostrum and Milk

Neonatal infection with HIV-1 has largely been prevented by ART prophylaxis during pregnancy [378]. Nevertheless, HIV-1 can be transmitted from mother to child via infected colostrum and milk particularly in resource limited countries [379]. Human colostrum and milk contain macrophages and DC that express DC-SIGN, which therefore could mediate HIV-1 *trans* infection of CD4^+^ T cells that are also present [379]. Interestingly, human milk can inhibit HIV-1 *trans* infection mediated by DC [380]. This occurs by binding of a bile salt-stimulated lipase in the Lewis X (LeX) glycoprotein found in human milk to DC-SIGN on the DC [381]. Human milk can also block HIV-1 *trans* infection through functional natural IgA and IgG antibodies specific for the carbohydrate recognition domain of DC-SIGN [382]. Strategies to block HIV-1 *trans* infection in breast milk include stimulation of TLR3 expressed on breast milk macrophages with its double-stranded RNA ligand poly (I:C) [383]. This process may work through the inhibition of DC-SIGN expression by IFN-*α* and IFN-*β* that are induced by TLR3 activation.

## 11. HIV-1 *Trans* Infection Mediated by B Lymphocytes

Although B lymphocytes are highly activated and modified beginning in the earliest phases of HIV-1 infection, they are not considered a significant, major target for HIV-1 *cis* infection [384]. Indeed, B cells are difficult to infect with HIV-1 in that they do not express CD4 or CCR5, although they express CXCR4. Nevertheless, B cells derived either from lymphoid tissue or from the peripheral blood of HIV-1-infected persons carry replication-competent X4 or R5 virus [384, 385]. Moreover, binding of HIV-1 immune complexes to CR2 or CD21 on the surface of B cells has been linked to subsequent *trans* infection of T cells [386–390]. These early studies therefore established that there is transmission of HIV-1 from B cells to T cells involving virus trapped in immune complexes on the surface of the B cells. This is similar to the trapping of HIV-1-antibody complexes by Fc and complement receptors on follicular dendritic cells (FDC) that reside in follicles of secondary lymphoid tissues [391]. Virus infectivity can be maintained on the FDC for months. The FDC serve as a reservoir for HIV-1 that can be genetically diverse from virus in other body compartments [392]. CD4^+^ T cells trafficking through the lymphatics are infected by the FDC-bound virus, with virus replication being driven by TNF-*α* produced by the FDC [393].

In 2006, two laboratories discovered that DC-SIGN is expressed on peripheral blood and lymphatic tissue B cells [394, 395], in particular after stimulation with surrogates of activated CD4^+^ T cells, that is, CD40L and IL-4 [395]. This CLR acts as a portal for endocytosis of both X4 and R5 HIV-1 that does not involve complement receptors or immune complexes [395]. Soon after binding of virus to DC-SIGN, the CLR is internalized and temporarily absent from the B cell surface. This *cis* infection is non-productive, yet virus can retain its infectivity for at least 2 days within the B cells. Interestingly, most of the captured virions are destroyed in B cell lines engineered to express DC-SIGN [192, 216]. However, a portion of the input virus survives in these cells and can rapidly *trans* infect T cells [183, 192, 396]. 

Comparable to DC-SIGN-expressing B cell lines, activated blood and tissue-derived B cells are highly efficient in mediating HIV-1 *trans* infection of CD4^+^ T cells [395]. As with DC, HIV-1 *trans* infection mediated by B cells can be blocked with anti-DC-SIGN mAb as well as mannan. Although B cells express high levels of CXCR4, treatment with anti-CXCR4 mAb prior to addition of HIV-1 to B cells does not inhibit *trans* infection of the T cells. Thus, although *cis* infection of B cells does not involve productive replication of infectious virions like that of DC and monocytes/macrophages, B cells are as efficient as these other professional APC in mediating HIV-1 *trans* infection. This DC-SIGN dependent process of B cell mediated *trans* infection is not unique to HIV-1, as a similar pathway has been reported for hepatitis C virus *trans* infection of hepatoma cells [397].

CD40L is expressed on activated CD4^+^ T cells, which are abundant throughout chronic immune activation that is prevalent in HIV-1 infection. CD40 is a normal constituent on B cell surfaces. Thus, the continuous interaction of CD40L on activated T cells with CD40 on B cells is one pathway of chronic B cell stimulation during HIV-1 infection [384]. Intriguingly, the hyperactive state of B cells in HIV-1 infection has also been linked to CD40L incorporated in HIV-1 virions [398]. Such virions are found in plasma of untreated HIV-1 infected patients and induce Ig as well as IL-6 and other cytokines and chemokines in B cells [399]. Importantly, host-derived CD40L within virions results in increased virus attachment to B cells and more efficient B cell mediated *trans* infection of autologous CD4^+^ T cells compared to virions without CD40L [398]. 

An emerging concept in HIV-1 *cis* and *trans* infection is the role of cross-talk among DC, B cells and other cell types. Thus, HIV-1 *cis* infection of DC is enhanced by coculture with CD4^+^ T or B lymphocytes but not with CD8^+^ T cells [400]. In addition to potential involvement of CD40-CD40L-related B cell activation in this process, an unexplored area of HIV-1 *cis* and, hence, *trans* infection is the role of the tumor necrosis factor receptor superfamily [401]. Specifically, B cell activating factor (BAFF) and proliferation-inducing ligand (APRIL) could be central to HIV-1 interaction with B cells. Interestingly, these factors are produced by DC and function to drive B cell differentiation and survival by both T cell dependent and independent pathways. Thus, HIV-1 infection could act through DC to hyperactivate B cell responses [402], which in turn could affect both HIV-1 *cis* and *trans* infection mediated by B cells. Another area of potential interest regarding HIV-1 *trans* infection mediated by B cells is a possible role of TLR both *in vitro* and *in vivo* [403]. For example, autoimmune complexes, which are highly prevalent during HIV-1 infection, can stimulate B cells via TLR7 and TLR9 [404]. TLRs have been associated with B cell activation during HIV-1 infection [384]. These factors need to be assessed in B cell models of HIV-1 *cis* and *trans* infection. 

B cells have also been implicated as intermediaries in enhanced HIV-1 infection of CD4^+^ T cells by macrophages [405]. This process involves activation of macrophages via Nef and CD40 ligation, leading to production of soluble CD23 and ICAM. These proteins act on B cells to upregulate expression of CD22, CD58, and CD80, which in turn stimulate CD4^+^ T cells via CD45, CD2, and CD28, respectively. The final result is enhanced HIV-1 replication in the T cells.

## 12. Concluding Remarks: Role of HIV-1 *Trans* Infection in HIV-1 Disease Progression

Despite years of extensive studies on HIV-1 *trans* infections, there are still major gaps in our understanding of this phenomenon. In particular, there is a paucity of information on the importance HIV-1 *trans* infection in natural HIV-1 infection and disease. There are inherent difficulties and highly challenging complexities in proving that this process is involved in virus transmission and disease pathogenesis. Nevertheless, studies of *trans* infection would be of interest in persons who control HIV-1 infection in the absence of ART, who range in definition and terminology as long-term nonprogressors, viral controllers, and elite controllers [406–408]. Myeloid DC from the blood of elite controllers have increased antigen presenting capacity and secrete less proinflammatory cytokines than HIV-1 infected progressors [409], which could affect both HIV-1 *cis* and *trans* infection. However, APC from these individuals have not been studied for their ability to *trans* infect T cells. We can speculate that the efficiency of this process will depend in part on the integrity of functional activity of the APC and CD4^+^ T cells, that is, their expression of receptors and intracellular and cell-cell pathways known to be involved in both HIV-1 *cis* and *trans* infections.
